# Cortical multisensory integration—a special role of the agranular insular cortex?

**DOI:** 10.1007/s00424-020-02400-6

**Published:** 2020-05-26

**Authors:** Daniela Brunert, Markus Rothermel

**Affiliations:** Department of Chemosensation, AG Neuromodulation, Institute for Biology II, RWTH Aachen University, 52074 Aachen, Germany

Multisensory integration describes the act of combining sensory information from different sources to form a cohesive real-world picture. It is considered one of the fundamental functions of the brain [3], but its exact mechanisms still pose an unresolved question in modern neuroscience. No matter if we watch TV, cross a road, eat lunch, or spend time with a beloved person, we experience most of our activities with more than one sensory modality, demonstrating the everyday importance of multisensory integration. How much we rely on its function is especially noticeable in instances when a multisensory mismatch creates problems e.g. if the audio feed in a video call is delayed or inconsistent signals between visual and vestibular input systems create motion sickness (e.g. virtual reality cyber-sickness).

One of the most complex examples of multisensory integration is flavor perception. Flavor not only involves the combination of gustatory, olfactory, and trigeminal inputs but also visual, auditory, and somatosensory stimuli contribute to flavor perception [10]. Where these modalities, especially gustation and olfaction, are combined to form the perception of a certain flavor is still not fully understood. Multiple sites have been implicated so far including the insula, operculum, caudal orbitofrontal cortex (OFC), and anterior cingulate cortex, theorized to form a kind of “flavor network” [6]. It is currently under debate if the OFC is the first area capable of responding to both olfactory and gustatory stimuli or if already primary olfactory and gustatory cortices themselves participate in the processing of multimodal chemosensory information [5].

In this issue of the *Pflügers Archiv - European Journal of Physiology*, Mizoguchi et al. present novel data on how the insular cortex, including the primary taste cortex, could be involved in the integration of olfactory and gustatory cues. The authors used voltage-sensitive dye (VSD) imaging to examine spatiotemporal dynamics in taste and olfactory cortical areas following chorda tympani (CT) nerve and olfactory bulb (OB) stimulation in the anesthetized rat. This is possible by using a preparational approach that allows the authors to visualize large parts of the cerebral cortex around the rhinal fissure and middle cerebral artery. This includes the insular and piriform cortex, the primary olfactory cortex. The authors recorded optical signals in parallel from these areas while stimulating the chorda tympani nerve or the olfactory bulb electrically in order to precisely control input timing and increase reliability. Chorda tympani stimulation elicited the largest VSD signals in the rostral dysgranular insular cortex (rDI) with smaller albeit significant signals detectable in the secondary somatosensory area/insular oral region but no signals in the piriform cortex. OB stimulation elicited the largest signals in the anterior and posterior piriform cortex but no signals in gustatory areas. Simultaneous stimulation did not change signals in gustatory or olfactory areas compared with stimulation of CT or OB alone except in the rostral agranular insular cortex (rAI). Here, unimodal signals seemed to be ineffective but simultaneous stimulation additively increased the measured signal amplitude. Based on these findings, the authors conclude that rather than in the primary sensory cortices, multisensory integration primarily happens in the rAI which is seen as part of the orbitofrontal cortex [4]. Thus, they confirm the findings of several human fMRI studies that highlighted the ventral insula as one of the areas that exhibit additive or even super additive effects to bimodal sensory presentation [1, 7–9]. Different publications have, however, shown gustatory responses in the piriform cortex [2] as well as olfactory responses in the gustatory cortex [5]. More studies are required to clarify how far orbitofrontal multisensory integration is distinct from multisensory responses in primary sensory and other lower brain areas.

In summary, Mizoguchi et al. contribute to elucidating the question of how the multimodal sense of “flavor” is processed, something essential if we want to understand the complex interplay of the factors controlling phenomena like cravings and hedonic value of food and ultimately, dietary choices, and the amount of food intake. It furthermore highlights the potential of optical imaging for the elucidation of multisensory integration giving a fast overview of cortical signals over relatively large areas.
